# Xp22.33 Duplication Encompassing PAR1 in a Male with Syndromic Neurodevelopmental Disorder and Tall Stature

**DOI:** 10.3390/genes17020238

**Published:** 2026-02-15

**Authors:** Dibyendu Dutta, Xi Luo, Ria Garg

**Affiliations:** 1 Division of Hematology and Oncology, Department of Medicine, State University of New York Upstate Medical University, Syracuse, NY 13210, USA; duttad@upstate.edu; 2 Department of Molecular and Human Genetics, Baylor College of Medicine, Houston, TX 77030, USA; xi.luo@bcm.edu; 3 Baylor Genetics, Houston, TX 77021, USA; 4 Center of Development, Behavior, and Genetics, State University of New York Upstate Medical University, Syracuse, NY 13210, USA

**Keywords:** Xp22.33 duplication, PAR1, *SHOX*, *DHRSX*, *CSF2RA*, *ASMT*, neurodevelopmental disorders, autism spectrum disorder, global developmental delay, tall stature

## Abstract

Background: Duplications involving Xp22.33, particularly within the pseudoautosomal region 1 (PAR1), are rare. While copy number variants (CNVs) involving *SHOX*, a dosage-sensitive gene in PAR1, are known to cause growth disorders, large duplications encompassing the entire PAR1 region and beyond show variable associations with skeletal and neurodevelopmental abnormalities. Duplication of the near-complete, isolated PAR1 with a comprehensive clinical description has not been reported. Case Presentation: We report a male patient with a 2.49 Mb duplication encompassing nearly the entire PAR1 region (chrX:200854–2692897, GRCh37). Clinical features included global developmental delay (GDD), autism spectrum disorder (ASD), recurrent seizures, hypotonia with joint hypermobility, dysmorphic features, and proportionate tall stature. The duplicated segment contains 30 genes, including 15 protein-coding genes that escape X-inactivation. Among these, *SHOX*, *DHRSX*, *ASMT*, and *CSF2RA* are notable candidates contributing to the observed phenotype. Conclusions: This report presents a detailed clinical characterization of a rare, near-complete, isolated PAR1 duplication in a male individual. The co-occurrence of tall stature, GDD, ASD, and seizures raises the possibility of a dosage-related phenotypic effect involving one or more genes within the duplicated interval. While causality cannot be definitively established, these observations contribute to the emerging understanding of the functional consequences of Xp22.33 duplications and suggest that increased copy number within this region may be associated with a clinically significant neurodevelopmental phenotype.

## 1. Introduction

Human sex chromosomes contain unique genomic intervals known as pseudoautosomal regions (PARs), located at the distal ends of the X and Y chromosomes. These regions undergo obligatory recombination during male meiosis to ensure proper chromosome segregation [1]. Two PARs are recognized in humans: PAR1, spanning approximately 2.6–2.7 Mb at Xp22.33/Yp11.32, and PAR2, a smaller region of around 320 kb at Xq28/Yq12. PAR1 is gene-rich and functionally significant, containing 15 protein-coding genes, including the dosage-sensitive short stature homeobox (*SHOX*) gene, as well as multiple conserved non-coding regulatory elements. Many PAR1 genes escape X-inactivation and are expressed from both X and Y alleles in males and from both X chromosomes in females. This results in intrinsic dosage sensitivity, especially in cases involving structural variation such as deletions or duplications, and highlights the critical role of PAR1 in early development and gametogenesis.

Among PAR1 genes, *SHOX* is the best characterized. *SHOX* haploinsufficiency causes idiopathic short stature (ISS) and Léri–Weill dyschondrosteosis, whereas duplications are associated with tall stature and more complex phenotypes, including Mayer–Rokitansky–Küster–Hauser syndrome [2,3,4,5,6]. *SHOX* expression is tightly regulated by cis-acting elements located upstream and downstream of the gene, and structural variants disrupting these regions are a well-established cause of growth disorders. For example, duplications encompassing *SHOX*-flanking regulatory elements, but not the gene itself, have been reported in patients with *SHOX* haploinsufficiency syndromes [7]. Conversely, large duplications spanning the full *SHOX* coding sequence and its enhancers can cause overexpression resulting in triplosensitivity and tall stature, while smaller duplications that disrupt cis-regulatory enhancer-promoter communication attenuate expression, resulting in short stature and congenital clubfoot [4,8].

Expansive duplications extending beyond PAR1 have been implicated in a broader clinical spectrum, including neurodevelopmental disorders, seizures, autism spectrum disorder (ASD), and congenital heart defects [9,10]. However, near-complete duplications encompassing almost the entire PAR1 region remain exceedingly rare, and detailed clinical descriptions are lacking. Here, we describe a male patient with a near-complete duplication of PAR1 who presented with severe global developmental delay (GDD), autistic features, recurrent seizures, generalized hypotonia with joint hypermobility, obesity, and tall stature. To our knowledge, this is the first reported case of an isolated, near-complete PAR1 duplication characterized with comprehensive clinical findings, which may offer new insights into the potential dosage-sensitive impact of PAR1 genes.

## 2. Case Presentation

The proband is a 3-year-old male referred for genetic evaluation due to GDD, ASD, a history of seizures, and multiple congenital anomalies. He was naturally conceived to a 36-year-old mother and a 33-year-old father, with no reported consanguinity. He was born at term via spontaneous vaginal delivery following an unremarkable pregnancy, aside from maternal hospitalization for COVID-19 infection in the third trimester. Apgar scores were 8 and 9 at one and five minutes, respectively. Birth measurements included weight 3.63 kg (57th percentile), length 53.3 cm (89th percentile), and head circumference 35.6 cm (45th percentile). There were no neonatal complications, and newborn hearing and cardiac screenings were passed.

Early infancy was notable for emergency department visits at 3 and 6 weeks of age for fever and congestion, both of which resolved without sequelae. Between 3 and 22 months of age, he experienced several common pediatric conditions, including recurrent upper respiratory symptoms, febrile illnesses, and viral infections such as bronchiolitis, hand-foot-and-mouth disease, and croup, the latter of which required dexamethasone treatment at 12 months. While he encountered two episodes of acute otitis media and a febrile respiratory illness with wheezing, all cases were managed with supportive care or antibiotics and resolved without the need for hospitalization or intravenous therapy. Additionally, his early medical history was notable for congenital nasolacrimal duct obstruction and pseudostrabismus, both managed expectantly, as well as chronic constipation that necessitated ongoing intervention with dietary modifications and polyethylene glycol.

**Figure 1:**
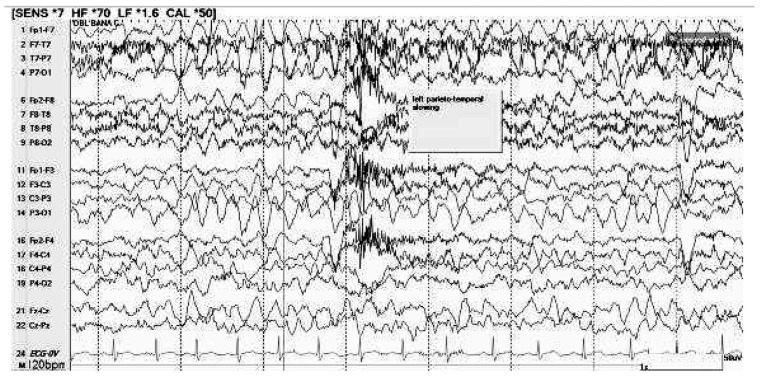
Abnormal electroencephalogram (EEG) in wakefulness due to intermittent delta slowing in the left temporo-parietal regions.

At 18 months, he experienced a generalized tonic–clonic seizure characterized by limpness, eye-rolling, and generalized twitching lasting between 30 and 60 s. Daily antiseizure medication was not initiated. Since the first noted event, caregivers reported intermittent staring spells, which were evaluated by neurology and considered behavioral rather than epileptic in nature. Electroencephalogram (EEG) at 19 months showed slowing in the left temporo-parietal region (Figure 1). While brain magnetic resonance imaging (MRI) at 22 months demonstrated T2 FLAIR hyperintensities in the bilateral periventricular white matter, most pronounced in the periatrial regions, mild splenial atrophy of the corpus callosum, and a left frontal horn choroid plexus cyst, the findings were considered nonspecific and age-appropriate. Seizure management consisted of ongoing clinical surveillance.

Developmental gross motor milestones were achieved on time: independent sitting by 6 months, cruising by 9 months, and walking at 14–15 months. However, speech development was significantly delayed. First words emerged between ages 2 and 3 years, and no two-word combinations were present at the time of evaluation. Hypotonia was noted in early childhood and improved over time, though joint hypermobility persisted. Fine motor development was mildly delayed, as he had not yet acquired a pincer grasp or independent utensil use. Social development was marked by limited peer interaction, absence of cooperative play, and self-injurious behaviors such as head-smacking. These behavioral features, together with stereotypies, limited expressive language, and poor social reciprocity, were consistent with ASD. He had not yet been toilet-trained. He was evaluated by early intervention and school-based services but is not actively receiving any therapy.

**Figure 2:**
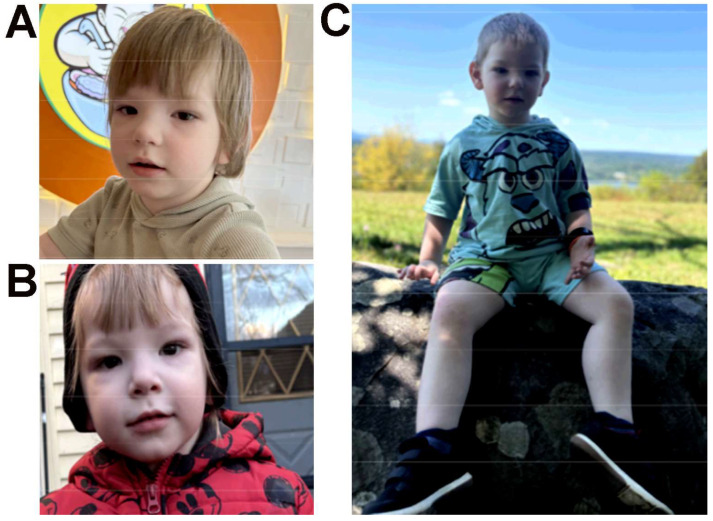
(**A**,**B**) Dysmorphic facial features showing prominent epicanthal folds, hypertelorism, deep-set eyes, a wide nasal base with depressed bridge and broad tip and (**C**) tall stature, larger ears.

On physical examination, dysmorphic features included brachycephaly, deeply set and widely spaced eyes with epicanthal folds, a depressed nasal bridge with a wide nasal base and broad nasal tip, widely spaced teeth, and ears that appeared large in size (Figure 2A,B). The patient also demonstrated generalized obesity, proportionate tall stature (Figure 2C), short toes, and joint hypermobility. Growth parameters at the most recent assessment were height 105.5 cm (>98th percentile), weight 20.1 kg (>99th percentile), and head circumference 49 cm (31st percentile), consistent with proportionate tall stature and generalized obesity. Neurologic findings included a history of hypotonia (improved over time) and persistent joint hypermobility. Additional concerns included congenital nasolacrimal duct obstruction, constipation managed with polyethylene glycol, and pseudostrabismus. Audiologic evaluation revealed normal behavioral thresholds and normal otoacoustic emissions.

### 2.1. Family History and Pedigree

**Figure 3:**
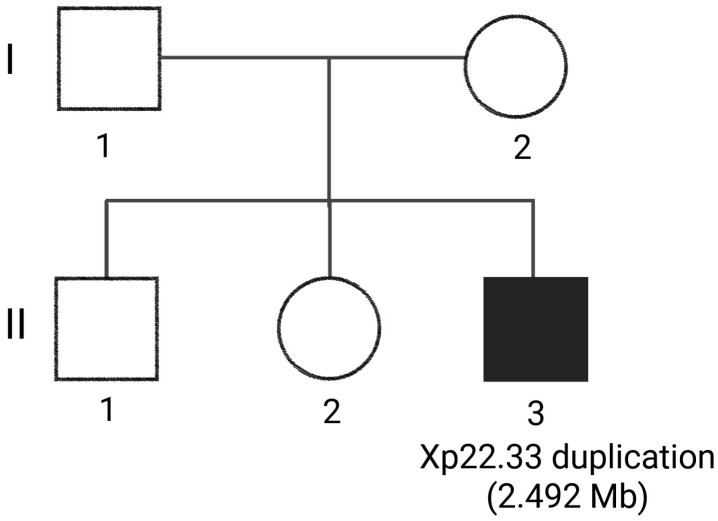
Family pedigree of the proband. I.2: Autoimmune disorder; II.1: 17 years old with autism spectrum disorder; II.2: 16 years old with autistic features; II.3: The proband was found to have a 2.492 Mb duplication at Xp22.33. The father (I.1) and siblings (II.1 and II.2) were not available for clinical evaluation or whole exome sequencing.

The family history of the proband is significant for neurodevelopmental concerns in both full siblings, including a 17-year-old brother diagnosed with ASD and a 16-year-old sister with autistic features (Figure 3). The mother is a 36-year-old female with a history of unspecified autoimmune conditions; her lineage includes a father with chronic obstructive pulmonary disease and a deceased mother with a history of both heart failure and chronic obstructive pulmonary disease. The father of the proband, a 33-year-old male, is reported to be alive and well, but is not available for testing or evaluation. His paternal history is more limited; it is notable for a deceased grandmother, a grandfather who suffered a myocardial infarction at age 65, and a 34-year-old paternal uncle with a history of seizures and epilepsy. Both parents report White ancestry, and there is no known consanguinity in the family.

### 2.2. Exome Sequencing (ES) and Variant Calling

Duo ES was pursued with his mother to investigate a suspected syndromic neurodevelopmental disorder in the proband. Informed consent was obtained for genetic testing and inclusion in this report. Testing was performed at Baylor Genetics (Houston, TX, USA).

Genomic DNA was fragmented, indexed, and enriched using exome probes to generate libraries, which were pooled and sequenced on an Illumina platform with paired-end reads. Sequencing data were aligned to the human reference genome (GRCh37/HG19) using the Illumina Dragen BioIT Platform, with variants called through its haplotype-based and CNV detection pipelines, modified by Baylor Genetics. Variant annotation and interpretation were conducted using automated platform of Emedgene with proprietary algorithms, incorporating population databases gnomAD, Exome Variant Server (EVS), ClinVar, and Human Gene Mutation Database (HGMD) Pro. All findings were interpreted in accordance with the American College of Medical Genetics (ACMG) guidelines [11,12] and reviewed in the context of the patient phenotype.

### 2.3. Genetic Findings

The proband was found to have a heterozygous 2.492 Mb duplication at Xp22.33 (chrX:200854–2692897, GRCh37) classified as a variant of uncertain significance (VUS) due to lack of previous published evidence for similar duplication in this region. The Xp22.33 duplication classification totaled a maximum of 0.6 points, which falls below the 0.9-point threshold required for a likely pathogenic designation under ACMG/ClinGen technical standards. In the initial assessment of genomic content (Sections 1–3), the duplication received 0 points. While the region contains *SHOX*, this gene maintains a ClinGen triplosensitivity score of 0, and no other established triplosensitivity genes are present. Additionally, the duplication does not disrupt any known haploinsufficiency genes and encompasses only 15 protein-coding genes, failing to meet the 35-gene threshold for scoring in Section 3. Clinical evidence evaluation (Section 4) contributed a maximum of 0.3 points based on rare reports of similar duplications in genomic databases. Although Xp22.33 duplications (PAR1) have been associated with clubfoot, this phenotype was not observed in the current patient. Other database entries, including DECIPHER (ID: 307724) and ClinVar (IDs: 686939, 1808677, 564679), provided limited clinical data or inheritance status, restricting the points assigned for phenotypic matching to 0.1. Finally, 0.1–0.3 points were assigned in Section 6, as the duplication has not been confirmed de novo.

The classification may be revised to likely pathogenic if future evidence emerges. This includes confirmation of a de novo origin (increasing Section 5 to 0.45 points) or the acquisition of more granular clinical data from existing database cases to strengthen phenotypic correlation. Furthermore, future population-based research or the discovery of novel triplosensitivity genes within the region may provide sufficient evidence to meet the pathogenicity threshold.

The duplicated region encompasses PAR1, which includes 30 genes of which 15 are protein-coding genes, several lncRNAs, miRNAs, and pseudogenes. The complete list of all the genes in the duplicated region along with their GRCh37 coordinates and a brief functional description is mentioned in Supplementary Table S1. Three genes within the duplicated region are listed in Online Mendelian Inheritance in Man (OMIM) with disease association: *SHOX* (OMIM 312865), associated with skeletal anomalies; dehydrogenase/reductase X-linked (*DHRSX*; OMIM 400049), an oxidoreductase expressed in brain and testis with suspected neurodevelopmental involvement; and colony stimulating factor 2 receptor subunit alpha (*CSF2RA*; OMIM 306250), which encodes a receptor subunit involved in immune and hematopoietic pathways. This duplication is absent in gnomAD or Database of Genomic Variants (DGV) and overlaps with CNVs reported in ClinVar and DatabasE of genomiC varIation and Phenotype in Humans using Ensembl Resources (DECIPHER) [13], including pathogenic and VUS classifications (Table 1).

### 2.4. Management

Following the identification of a partial duplication at Xp22.33, classified as a VUS, a comprehensive, symptom-based management strategy was established to address the complex multisystem needs of the proband. While a single generalized tonic–clonic seizure was noted, the lack of recurrent events and specific findings on EEG, which showed intermittent left temporoparietal delta slowing, and brain MRI, with nonspecific findings led to a recommendation for clinical monitoring and seizure precautions rather than immediate antiseizure medication. A repeat MRI at an older age was recommended to accurately evaluate the findings. To support significant GDD and autistic features characterized by minimal language, sensory sensitivities, and hypotonia, the proband is engaged in a multidisciplinary therapy regimen including speech, occupational, and physical therapies. Further referral was made to Developmental and Behavioral Pediatrics for behavior analysis for autism-related features, and planning for educational supports, including individualized education program evaluation at school entry. Early Intervention services were pursued initially, with transition to the Committee on Preschool Special Education; therapy access has been intermittently limited by system-level delays, though participation continues when available, with gradual developmental progress. Associated medical concerns, such as constipation, feeding difficulties, and dental enamel staining, are managed through conservative interventions like polyethylene glycol and nutritional counseling, while a cardiology evaluation confirmed normal cardiac structure and function without the need for ongoing follow-up. Furthermore, as the infection history of the proband remains within typical pediatric limits and does not suggest immunodeficiency, formal immunologic testing is deferred in favor of a scheduled genetic reassessment in two years to re-evaluate the Xp22.33 variant as more clinical data emerges.

Genetic counseling was provided to the mother regarding the Xp22.33 duplication, which currently remains classified as a VUS. It was mentioned that testing of the siblings and the father may reclassify the VUS. While maternal testing did not identify the duplication, paternal testing was unavailable, and laboratory results have not yet definitively clarified whether the duplication is located on the X or Y chromosome; consequently, precise recurrence-risk counseling cannot be provided at this time. It was explained that if future testing confirms the event is de novo, the recurrence risk is expected to be low, though the possibility of parental germline mosaicism prevents it from being zero. Conversely, if the variant is found to be inherited, the risk would depend on the parent of origin and, if X-linked, may vary by fetal sex. Because the finding remains a VUS, the family was counseled that it should not be utilized for clinical decision-making or as a basis for targeted prenatal diagnosis unless it is reclassified in the future. The pedigree (Figure 3) demonstrates that both siblings have neurodevelopmental findings, including ASD in the brother and autistic features in the sister. Although segregation analysis could strengthen the genotype–phenotype correlation, testing remains incomplete. If the Xp22.33 duplication were confirmed in the affected siblings, this would support a potential contributory role.

PAR1 is shared between the X and Y chromosomes and spans approximately 2.6–2.7 Mb at the terminal short arms. Genes within PAR1 generally escape X-inactivation, resulting in two functional gene copies at baseline in both males (one copy on X and one on Y) and females (one copy on each X chromosome). A single duplication involving PAR1 is therefore expected to increase gene dosage to approximately three copies, regardless of whether it occurs on the X or Y chromosome in males or on one X chromosome in females. Therefore, the presence of the duplication in either sibling would primarily inform segregation and inheritance patterns rather than sex-specific dosage effects.

## 3. Discussion

We report a male proband with a near-complete duplication of the PAR1 region at Xp22.33, presenting with severe GDD, ASD, recurrent seizures, dysmorphic features, hypotonia with hypermobility, and proportionate tall stature with obesity. Previously published cases involving duplications in this region either extended beyond PAR1 or lacked a complete characterization (Table 1). We explored the possible association between duplication of this genomic region and neurodevelopmental delay.

While the proband was prenatally exposed to maternal SARS-CoV2 infection, causality cannot be established, and its contribution to the proband’s neurodevelopment remains uncertain. Recent large cohort data suggest a potential increased risk of early childhood neurodevelopmental diagnoses following prenatal SARS-CoV2 infection, particularly with third-trimester exposure [14]. In contrast, other prospective studies using validated developmental screening tools have not identified significant associations through two years of age [15,16], underscoring the need for continued longitudinal follow-up. An important limitation across these studies is the absence of systematic genetic evaluation of affected children. Comprehensive genetic testing, including chromosomal microarray and/or exome sequencing, is considered standard clinical practice in the evaluation of children with developmental delay or neurodevelopmental disorders. Therefore, underlying genetic etiologies in reported cases cannot be excluded, and prenatal SARS-CoV2 exposure should be interpreted cautiously as a potential contributing rather than definitive causal factor [17].

Genes in the duplicated interval escape X-inactivation and undergo obligatory recombination with the Y chromosome during male meiosis. Because PAR1 genes are expressed from both alleles in males and females, duplications in males are particularly dosage-sensitive. Unlike females, in whom X-linked dosage is partly buffered by X-inactivation, hemizygous duplications in males can result in unopposed gene overexpression and more severe phenotypic consequences [18]. In sex chromosome aneuploidies such as Klinefelter syndrome (47,XXY), the increased dosage of genes that escape X-inactivation, particularly those within the PAR1, has been heavily implicated in various neurodevelopmental and cognitive phenotypes. Transcriptomic studies across multiple tissues consistently reveal an enrichment of these *escape genes* among dosage-sensitive, X-linked differentially expressed genes. Furthermore, experimental models, including induced pluripotent stem cells, demonstrate that PAR1 gene expression scales proportionally with sex chromosome dosage, triggering broader downstream transcriptomic effects. Despite these findings, current human data remain insufficient to quantify the specific contribution of PAR1 gene dosage to neurodevelopmental outcomes. This limitation arises because existing studies cannot yet disentangle PAR1-specific effects from the influences of other escape genes, whole-chromosome dosage, hormonal variations, and complex epigenetic factors.

The PAR1 region contains approximately 30 functionally diverse genes, which can be broadly categorized into five groups based on gene ontology, OMIM, and ClinGen annotations. For example, *SHOX* is implicated in skeletal development; *DHRSX*, acetylserotonin O-methyltransferase (*ASMT*), and acetylserotonin O-methyltransferase-like (*ASMTL*) have roles in neurodevelopment and brain function; while *CSF2RA* contributes to immune regulation. A list of all the functional grouping of the genes in the duplicated region is mentioned in Supplementary Table S2. For instance, *SHOX* and *PPP2R3B* have a potential role in growth and skeletal development, where *SHOX* regulates growth plate function, and *PPP2R3B* have a role in cell cycle and skeletal pathways.

### 3.1. Genes That May Contribute to Xp22.33 Duplication Phenotypes

#### 3.1.1. SHOX

*SHOX* encodes a homeobox transcription factor expressed during both embryonic and fetal development, particularly in the developing limb buds and the first and second pharyngeal arches [19]. It plays a critical role in skeletal development. During early limb formation, *SHOX* is initially expressed in undifferentiated mesenchymal cells. As mesenchymal condensations form and chondrogenesis begin, expression becomes enriched in the perichondrial layer-the site of chondroblast and subsequent osteoblast differentiation that establishes the cartilaginous framework surrounding the condensation. This spatially restricted expression underscores a direct role for *SHOX* in regulating chondrocyte differentiation and bone morphogenesis [19].

Duplications of *SHOX* have been associated with both tall and short stature, depending on the extent of the duplicated region. Multiple studies have demonstrated that duplications encompassing the entire *SHOX* coding sequence together with its cis-acting enhancers are linked to tall stature, consistent with *SHOX* overexpression [20,21,22]. Duplications of the complete *SHOX* gene and regulatory landscape are predicted to result in triplosensitivity. This mechanism parallels observations in individuals with sex chromosome aneuploidies (47,XXX; 47,XXY; 47,XYY), in whom the presence of an additional *SHOX* allele may have contributed to increased linear growth [20]. By contrast, partial duplications restricted to regulatory elements or only the gene may disrupt cis-regulatory architecture, leading to attenuated *SHOX* expression and haploinsufficiency syndromes characterized by short stature [4]. Indeed, clinical outcomes of tall stature, normal height, or short stature was reported to be dependent on duplication size, genomic location, and whether regulatory non-coding elements are included or structurally altered [2,23]. Proposed mechanisms underlying this variability include disruption of enhancer-promoter interactions, alterations in chromatin architecture, and increased genomic distance between *SHOX* and its regulatory elements, all of which may reduce functional *SHOX* expression despite copy number gain [23].

Aberrant *SHOX* expressions in the pharyngeal arches may also underlie craniofacial anomalies. The mesenchyme of the first pharyngeal arch contributes to the maxilla, mandible, and portions of the external and middle ear skeleton, while the second arch gives rise to additional skeletal structures, including components of the middle ear [19]. Overexpression of *SHOX* during development of these structures could perturb maxillomandibular growth, providing a plausible explanation for the widely spaced teeth observed in the proband.

##### *DHRSX* 

*DHRSX*, a member of the short-chain dehydrogenase/reductase (SDR) superfamily, is a multifunctional enzyme that acts as both a reductase and a dehydrogenase in dolichol biosynthesis. It serves two complementary functions critical for neurodevelopment: as a key enzyme in dolichol-dependent N-glycosylation, necessary for proper folding and trafficking of synaptic proteins including ion channels, neurotransmitter receptors, and adhesion molecules, and as a non-classical secretory protein that positively regulates starvation-induced autophagy, a process essential for neuronal progenitor survival, neurite pruning, and synaptic remodeling [24]. Dysregulation of *DHRSX,* therefore, may impair glycoprotein processing and autophagic homeostasis during neural development.

Hypoglycosylation of synaptic proteins may disrupt the excitatory-inhibitory balance, impair synaptogenesis, and compromise neurotransmission, leading to cognitive deficits and behavioral phenotypes overlapping with ASD. CNVs across PAR1 further implicate dosage imbalance in neurodevelopmental risk; for instance, overexpression of PAR1 genes, including *DHRSX*, alters the transcriptome of induced pluripotent stem cells (iPSCs) derived from sex chromosome aneuploidies [25]. These findings may support the notion that *DHRSX* dysregulation, whether due to loss-of-function variants or CNV dosage effects, may contribute to the clinical phenotypes observed. Indeed, biallelic missense variants in *DHRSX* have been associated with GDD, intellectual disability, ASD, seizures, hypotonia, craniofacial dysmorphism, and obesity [26,27]. Similarly, PAR1 CNVs in sex chromosome aneuploidies, such as Klinefelter syndrome, Jacob syndrome (XYY), and higher-grade aneuploidies (48,XYYY and 49,XYYYY), are associated with overlapping neurodevelopmental traits, including delayed speech and language development, attention-deficit/hyperactivity disorder (ADHD), intellectual disability, and ASD [28,29,30,31,32,33,34].

Neurological features such as epilepsy and hypotonia are also recurrent in glycosylation disorders [24]. Individuals with missense *DHRSX* variants frequently present with seizures and hypotonia, consistent with this mechanism [26]. Craniofacial anomalies are another common manifestation, reflecting the dependence of neural crest cell migration and extracellular matrix interactions on glycosylated receptors and adhesion molecules. Integrin α4, which contains multiple N-linked glycosylation sites, is expressed in neural crest cells and mediates essential cell–cell and cell–matrix adhesion during development [35]. Consistent with these mechanisms, dysmorphic facial features are often observed in individuals with *DHRSX* missense variants [26].

##### *ASMT* and *ASMTL*

*ASMT* is highly expressed in the pineal gland and thymus [36,37]. It encodes the enzyme that catalyzes the final step of melatonin biosynthesis, converting N-acetylserotonin into melatonin. Melatonin is not only a circadian rhythm regulator but also a neurotrophic and neuroprotective molecule during embryonic and fetal brain development [38]. Melatonin influences proliferation, differentiation, and survival of neuronal progenitors, and modulates synaptogenesis and dendritic growth [39]. Altered melatonin levels during embryogenesis and early postnatal life have been linked to disrupted sleep–wake cycles, impaired neuronal maturation, and increased vulnerability to neurodevelopmental disorders. Importantly, *ASMT* variants and reduced enzyme activity have been associated with ASD and intellectual disability in multiple cohorts [40,41,42].

*ASMTL* is a fusion gene composed of two distinct genes of different evolutionary origins and functions. The C-terminal region of the ASMTL protein contains a methyltransferase domain identical to that of ASMT, while the N-terminal region shows strong similarity to the multicopy-associated filamentation (maf) protein of *Bacillus subtilis* and to orfE of *Escherichia coli*, gene products involved in regulating cell division and growth [43]. A 270 kb duplication at Xp22.33 encompassing *CRLF2*, *CSF2RA*, *SLC25A6*, and *ASMTL* has been associated with ASD [44]. In a large-scale protein interaction study defining the Autism Spliceform Interaction Network (ASIN), *ASMTL* was identified as an ASIN gene and genetic risk factor for autism, shown to interact with tryptophan 2,3-dioxygenase (*TDO2*), an autism candidate gene [42].

### 3.2. Other Genes of Interest

Although the function of other PAR1 genes is not well characterized in the development context, dysregulation of *CSF2RA*, solute carrier family 25, member 6 (*SLC25A6*) and *CD99* has been associated with clinical phenotypes. For example, the cell surface receptor encoded by *CSF2RA* binds to the granulocyte/macrophage colony-stimulating factor (CSF2) on hematopoietic lineages. CSF2 signaling is required for pulmonary alveolar macrophage catabolism of surfactant. Dysregulation of *CSF2RA* leads to the excessive accumulation of surfactant-derived lipoproteins within pulmonary alveoli, causing severe respiratory distress and pulmonary alveolar proteinosis (PAP) [45,46]. Dysregulation of *SLC25A6* and *CD99* has been linked to frequent Klinefelter syndrome traits, such as cardiac abnormalities, intellectual disability [47,48], and autoimmune diseases [49,50].

## 4. Limitations

The duplication in the proband cannot be definitively identified as the sole cause of the pathogenic phenotype, nor can the influence of undetected variants be excluded. Limitations of this report include the unavailability of the biological father and lack of genetic evaluation of affected siblings, precluding determination of inheritance, segregation analysis, and assessment of intrafamilial variability. The duplication maps to PAR1, where shared X-Y homology prevents assignment of chromosomal origin or parental inheritance using standard CNV analysis, limiting interpretation to gene dosage effects. Furthermore, the performance of supporting or complementary research-level testing is often precluded by clinical constraints. Consequently, the direct causal link between a gene and a phenotypic condition is primarily established through literature-based evidence. Although sleep disturbances are frequently associated with ASD, and *ASMT* is involved in melatonin synthesis, that regulates the sleep–wake cycle, its direct contribution to the behavioral phenotype in the proband remains uncertain due to the lack of melatonin measurements or objective sleep data. Bone age imaging was not performed because the patient initially presented to the Genetics clinic for developmental delay and dysmorphic features and was not referred to Endocrinology. Following genetic testing, tall stature was considered most consistent with *SHOX* duplication rather than a primary endocrine disorder and bone age assessment was not expected to alter clinical management in this case. Previous studies have demonstrated variable bone age findings in *SHOX*-related growth disorders; therefore, bone age evaluation may be considered when clinically indicated.

## 5. Conclusions

This report describes a near-complete PAR1 duplication in a proband with a syndromic neurodevelopmental phenotype. The co-occurrence of autism, seizures, and tall stature in a male with an isolated PAR1 gain suggests a possible dosage-related effect of genes within this region. These findings may help narrow the critical interval for neurodevelopmental involvement within PAR1 and further clarify the functional impact of Xp22.33 duplications.

## Figures and Tables

**Table 1:** ClinVar, DECIPHER and published cases with PAR1 duplications comparable to the proband.

Study	Coordinates (hg19)/Band	Size (Mb)	Gene Count	Phenotypes	Classification
Present Case	X:200854–2692897	2.492	30	Global developmental delay, regression, autism spectrum disorder, seizures, tall stature, dysmorphism	Uncertain significance
† Sadler et al., 2020 [8]	X:200854–2688632	2.487	30	Bilateral clubfoot, torticollis, developmental delay (had additional chr2q37.3 deletion)	NA
† Sadler et al., 2020 [8]	X:200854–2692780	2.491	30	Bilateral clubfoot	NA
† Sadler et al., 2020 [8]	X:200854–2692780	2.491	30	Bilateral clubfoot, bilateral camptodactyly	NA
† Sadler et al., 2020 [8]	X:200854–2692780	2.491	30	Bilateral clubfoot, developmental delay	NA
† Sadler et al., 2020 [8]	X:200854–2729493	2.528	30	Bilateral clubfoot, developmental delay (had additional chr16p13.11 duplication)	NA
DECIPHER 307724	X:61115–2709057	2.65	31	Clubfoot, developmental delay, motor stereotypy, dysmorphism, speech delay	Pathogenic
DECIPHER 285320	X:10140–2734771	2.72	31	Developmental delay, joint hypermobility	Uncertain significance
DECIPHER 421722	X:159430–2732910	2.57	31	Not provided	NA
ClinVar 686939	X:168546–2696762	2.528	30	Delayed milestones in childhood, intellectual disability, attention-deficit/hyperactivity disorder (ADHD)	Pathogenic
ClinVar 1808677	X:201705–2696762	2.495	30	Not provided	Pathogenic
ClinVar 564679	X:168546–2589564	2.421	28	Not provided	Uncertain significance
ClinVar 3024647	X:198048–2670376	2.472	30	Not provided	Uncertain significance

† Exome sequence data from only clubfoot cases analyzed. Note: ClinVar was queried for X-chromosomal copy number gains similar to the present duplication (GRCh37:chrX:200854–2692897). Using the GRCh37/hg19 assembly, 394 variants met inclusion criteria (67 Pathogenic, 5 Likely Pathogenic, 322 Uncertain Significance). Variants were ranked by similarity in genomic position and size. Only one CNV, ClinVar 1808677 (Pathogenic), matched within a <5 kb size difference and remained the closest match at <10 kb. Expanding the threshold to <50 kb identified four variants total, but ClinVar 1808677 consistently showed the highest similarity in both size and location.

## Data Availability

The data supporting these findings are available on request from the corresponding author. The data are not publicly available due to privacy or ethical concerns.

## Supplementary Materials

The following supporting information can be downloaded at: https://www.mdpi.com/article/10.3390/genes17020238/s1, Table S1: Full list of genes within the Xp22.33 duplication region (GRCh37); Table S2: Functional grouping of genes in Xp22.33 duplicated region.
