# Supplementary material for: Xp22.33 Duplication Encompassing PAR1 in a Male with Syndromic Neurodevelopmental Disorder and Tall Stature
Source: Genes (Basel). 2026 Feb 15;17(2):238. doi: 10.3390/genes17020238 (PMC12941262; doi:10.3390/genes17020238)
Supplement: Supplementary file 1 [file genes-17-00238-s001.zip › Supplementary Table S1.pdf]

Supplementary Table S1: Full list of genes within the Xp22.33 duplication region (GRCh37)

No.	Gene	Coordinates (hg19)	Description
1	GTPBP6	X:303,346–318,798	GTP-binding protein, brain/testis expression
2	PPP2R3B	X:333,933–386,955	Protein phosphatase regulatory subunit
3	PLCXD1	X:276,322–303,356	Phospholipase, signal transduction role
4	SHOX	X:624,344–659,411	Skeletal development gene
5	SLC25A6	X:1,386,152–1,392,113	Mitochondrial carrier
6	IL3RA	X:1,336,616–1,382,689	Cytokine receptor (IL-3 alpha)
7	CRLF2	X:1,190,490–1,212,723	Cytokine receptor-like factor
8	CSF2RA	X:1,268,793–1,325,373	Colony stimulating factor receptor
9	P2RY8	X:1,462,581–1,537,185	B-cell signaling receptor
10	ASMTL	X:1,403,139–1,453,762	Melatonin synthesis-related
11	ASMT	X:1,615,059–1,643,081	Melatonin pathway; associated with ASD
12	ZBED1	X:2,486,414–2,500,976	Transcription factor
13	DHRSX	X:2,219,506–2,502,805	Oxidoreductase; neurodevelopmental potential
14	CD99	X:2,691,187–2,741,309	Cell adhesion molecule
15	XG	X:2,752,040–2,816,500	Xg blood group protein
16	AKAP17A	X:1,591,604–1,602,520	A-kinase anchoring protein
17	CD99P1	X:2,609,415–2,623,453	CD99 pseudogene
18	DHRSX-IT1	X:2,334,295–2,336,410	Intronic transcript
19	FABP5P13	X:523,775–524,102	Fatty acid-binding pseudogene
20	KRT18P53	X:545,236–545,352	Keratin pseudogene
21	LINC00102	X:2,612,988–2,615,347	Long non-coding RNA
22	LINC00106	X:1,392,679–1,400,524	Long non-coding RNA
23	LINC00685	X:320,990–321,851	Long non-coding RNA
24	LINC02968	X:1,657,735–1,763,312	Long non-coding RNA

25	LINC03112	X:2,566,022–2,609,292	Long non-coding RNA
26	MIR3690	X:1,293,918–1,293,992	MicroRNA
27	MIR6089	X:2,609,191–2,609,254	MicroRNA
28	RNA5SP498	X:1,300,256–1,300,375	5S ribosomal pseudogene
29	RPL14P5	X:1,008,503–1,010,101	Ribosomal protein pseudogene
30	DHRSX-IT1	X:2,334,295–2,336,410	Intronic transcript (duplicate listing)
