# Supplementary material for: Xp22.33 Duplication Encompassing PAR1 in a Male with Syndromic Neurodevelopmental Disorder and Tall Stature
Source: Genes (Basel). 2026 Feb 15;17(2):238. doi: 10.3390/genes17020238 (PMC12941262; doi:10.3390/genes17020238)
Supplement: Supplementary file 1 [file genes-17-00238-s001.zip › Supplementary Table S2.pdf]

Supplementary Table S2: Functional grouping of genes in Xp22.33 duplicated region

Category	Genes	Potential Roles
**Neurodevelopment**	DHRSX, GTPBP6, ASMT, ASMTL, P2RY8, PLCXD1, ZBED1	CNS development, melatonin metabolism, synaptic signaling
**Growth/Skeletal**	SHOX, PPP2R3B	SHOX regulates growth plate function; PPP2R3B in cell cycle and skeletal pathways
**Immune/Metabolic/ Hematologic**	CSF2RA, CRLF2, IL3RA, P2RY8, SLC25A6, CD99, XG	Cytokine signaling, metabolic regulation, energy transport, cell adhesion
**Regulatory (lncRNA)**	LINC00102, LINC00106, LINC00685, LINC02968, LINC03112	Gene expression regulation, chromatin remodeling
**Regulatory (miRNA)**	MIR3690, MIR6089	Fine-tune gene expression post-transcriptionally
**Pseudogenes**	FABP5P13, KRT18P53, CD99P1, RNA5SP498, RPL14P5	Possible competing endogenous RNA activity; transcriptional modulation
**Other**	AKAP17A, DHRSX-IT1	Protein kinase anchoring, intronic transcripts with regulatory potential
